# Consequential (and inconsequential) environmental epidemiology

**DOI:** 10.1097/EE9.0000000000000433

**Published:** 2025-10-22

**Authors:** David A. Savitz, Gregory A. Wellenius

**Affiliations:** a Brown University School of Public Health, Providence, Rhode Island; b Boston University School of Public Health, Boston, Massachusetts

Epidemiologists have effectively exploited advances in assessing exposure and health outcomes to pursue potential causal links between the environment and disease. The rapidly decreasing cost of assaying biospecimens for multiple chemicals and the proliferation of biobanks from established surveys (e.g., the National Health and Nutrition Examination Surveys, https://www.cdc.gov/nchs/hus/sources-definitions/nhanes.htm), repositories (e.g., UK Biobank, https://www.ukbiobank.ac.uk/), and study cohorts (e.g., the MIREC Biobank, https://www.mirec-canada.ca/en/about) provide a vast array of research opportunities. Analogously, the assessment of health endpoints based on clinical biomarkers is often inexpensive for common measures such as hormones, lipids, or markers of inflammation. Although the designs vary somewhat, the prototypic study examines one or more exposure biomarkers (generally in the low range) in relation to one or more clinical biomarkers (typically in the subclinical range).

Although in principle, more evidence can only be beneficial, the rapid growth in the availability of comprehensive datasets coupled with lower barriers to analyzing those data (including through AI-assisted analyses and manuscript preparation) is leading to a deluge of new publications.^1^ Many of these studies identify some positive associations, often with reasonable statistical power, even in relatively small populations. However, given (a) the tenuous connection between environmental biomarkers and exposure sources, (b) the indirect relevance of subclinical health indicators to disease, and (c) abundant opportunities for both false positives and selective publication of positive results, it is not clear that the high volume of such analyses offers progress toward identification of important etiologic relationships or the improvement of public health.

## Disconnect between biomarkers and environmental exposures

Ideally, exposure biomarkers provide an integrated measure of internal dose of an environmental toxicant, circumventing the need to assess environmental sources or query individual behaviors. Given advances in technology, the menu of candidate exposure biomarkers is extensive, and for many exposures, nearly everyone has detectable levels. But for many chemicals, the connection between environmental sources and biomarker levels is ill-defined. For example, some forms of PFAS are present in virtually everyone, but except for individuals exposed to specific sources from contaminated drinking water, a unique dietary source, or through the work environment, the sources of exposure are unknown.

In populations with typical background exposures, differences in exposure biomarkers among study participants tend to be quite small and studies end up contrasting those with very low exposure to those with extremely low exposure. Measurement error alone generates interindividual differences, but differences due to random measurement error alone would be expected to underestimate any underlying causal effects. Metabolic variation in uptake, metabolism, and excretion among individuals with essentially the same exogenous sources can generate variation in exposure biomarkers. To the extent that metabolic variation drives variation in exposure biomarkers, spurious associations with health outcomes are likely to be found,^2,3^ unrelated to exogenous exposure.

Since the goal of etiologic research is to identify opportunities to reduce exposure and improve public health,^4^ biomarkers are problematic since they are not typically amenable to intervention; only the exogenous determinants of exposure can be changed. For the study to suggest actions to reduce exposure, it needs to address a specific exposure source, such as the concentration of a toxicant in air or water, or a behavioral influence on exposure, such as diet or use of a consumer product. In simple terms, if the goal of research is to identify causal effects of environmental exposures^5^ rather than to merely describe associations involving environmental agents, then studies based on biomarkers without a clear exogenous source are minimally helpful.

## Significance of health outcomes

Many studies of the potential health effects of environmental toxicants consider subclinical health outcomes, variation within the normal range, given their reliance on relatively small convenience samples. Commonly used health endpoints include clinical biomarkers (e.g., lipids, hormones, and micronutrients), anthropometric measures (e.g., birthweight and body mass index), physiological measures (e.g., blood pressure), and scales assessing symptoms, behaviors, or capability (e.g., neurobehavioral tests). By considering continuous measures, even small studies may have sufficient statistical power to detect associations, but the magnitude of difference in the health outcome across individuals with relatively low exposures is often clinically inconsequential.

The standard defense for such studies of variation in the normal range includes two claims: (1) a small change in a marker of health applied across a large population may have important public health consequences, even if the magnitude is not important for any individual, and (2) even if the effect is modest, some individuals will be pushed over a threshold such that their health is meaningfully worse as a result. If the small change is causal, and a sizeable population is affected, there would be public health relevance so long as there is clear, independent evidence of the presumed downstream consequences. For example, while studies of environmental toxicants may not be capable of demonstrating that a small increment in blood pressure results in elevated risk of stroke, since other studies have already established the relationship between blood pressure and stroke, even a small increase in average blood pressure is meaningful on a population level. In contrast, a small shift in thyroid hormones or birthweight may not have significant health consequences.

The second argument concerns exposure causing some individuals to cross a clinically consequential threshold. There are very few health phenomena that have real thresholds dividing subclinical alterations from clinically consequential changes, even though arbitrary cutpoints are frequently used to facilitate clinical diagnoses or risk stratification. Statistically, shifting the entire distribution of an outcome will change the proportion of the population falling above any given threshold, but that does not mean that even those individuals who cross the threshold are harmed. Going from a birthweight of 2505 to 2495 g would lead to a shift from “normal birthweight” to “low birthweight” but the threshold itself is arbitrary, and the 10 g shift is without health consequences. A similar argument can be made for defining obesity based on body mass index, diabetes or prediabetes based on categorization of levels of hemoglobin A1c, hypercholesterolemia, or hypertension. The main consequence of crossing the threshold is qualifying for clinical intervention.

## Pathways to false positive findings

Identifying small causal effects is challenging, requiring researchers to effectively distinguish between “no effect” and “a very small effect.” Many health outcomes have strong sociodemographic determinants and many (but by no means all) environmental toxicants are higher in socially disadvantaged groups. In this scenario, confounding by social factors is present, and even after attempts at statistical adjustment for social determinants, residual confounding is likely to remain. Constructs such as “social disadvantage” are extremely difficult to fully capture, with indicators such as education or income helpful but incomplete. Statistical control of confounding is only effective to the extent that the putative source of confounding is accurately measured. Biomarkers associated with various lifestyle factors and physiologic variation may be confounded in ways that cannot be fully addressed through conventional statistical approaches, calling for more sophisticated designs such as Mendelian randomization or the use of instrumental variables. Finding that an unadjusted association between an environmental agent and disease is markedly attenuated (but not eliminated) by adjustment for an imperfectly measured confounder suggests more complete adjustment would likely result in an even weaker association.^6,7^ Distinguishing between a small causal effect and an association with residual confounding may simply not be possible.

Studies of environmental biomarkers often address multiple chemicals and use a variety of metrics of exposure based on varying cutpoints or combinations of exposures. Health outcomes may include multiple options (e.g., scales of behavior and different hormones) as well as varying cutpoints or combinations of measures. Subgroups based on sex, ethnicity, calendar time, or other covariates may be evaluated, proliferating the candidate pool of associations. The potential for at least some false positives is substantial. However, the solution is not to make formal adjustments for statistical tests^8^ but instead to examine the coherence of the findings, considering prior research, strength of the association, presence of dose-response gradients, and other informative patterns in the data. Distinguishing between “blips” and “signals” is not purely a statistical issue but a conceptual one that needs to draw on subject matter knowledge, previous research, and patterns in the study data.

Use of flexible analytic approaches exacerbates the problem of false positives.^9^ In environmental epidemiology, splines are now easy to implement and allow the analyst to detect nonlinear or even nonmonotonic relationships. However, segments of the regression line that are positive somewhere in the range of exposure may fit the data well but lead to inexplicable and nonreplicable results. Similarly, when the study of individual exposures does not yield clear associations or readily interpretable results, various mixture models can be used to fit the data and generate positive associations but not point to modifiable causes of disease.^10^ In studies of chronic disease, there is an opportunity to evaluate multiple lag periods, increasing the opportunity to find positive associations.^11^ The problem is a combination of overfitting the data and a tendency to overinterpret the results.

When the goal is to find positive associations rather than accurately estimate causal effects (including null effects), then positive associations become “interesting” and null or negative associations are “uninformative.” A balanced interpretation of study results means that all results contribute information, including those that fail to support or are counter to the hypothesized association. Investigator bias is most often revealed in the abstract, results text, and in the summary of findings in the discussion; an essentially negative study with some possible exceptions is touted as a positive study based on any deviations from the null in a direction that supports an adverse effect of environmental toxicants.

## Recommendations for more informative studies

Generating actionable knowledge of environmental toxicants and health^12^ requires identification of promising research opportunities, not just exploiting readily available data. Studies of populations with a well-defined exposure source, even if exposure is measured imperfectly, have notably different strengths (and limitations) than studies of background levels of biomarkers.^2^ The range of exposures is often higher, the basis for exposure contrasts is clear, sources of potential confounding may be more amenable to control, and the implications for potential interventions are obvious. In that setting, exposure biomarkers may help to validate exposure contrasts even if they are not the primary exposure indicator.

Evaluating the health relevance of small, subclinical effects may benefit from considering evidence from other disciplines, including toxicology, clinical research, and mechanistic studies to help determine whether the observed effects are, in fact, pathological in nature. For example, suitable animal models for assessing fetal growth or endocrine disruption may be applicable to judging whether subclinical biological changes observed in epidemiologic studies have relevance to clinically consequential health outcomes. Triangulation of evidence^5^ may help to determine whether subclinical effects observed in epidemiologic studies have important health consequences.

Toxicological or mechanistic research may also help to circumvent the challenge of distinguishing between very small effects and no effect. Identifying more potent forms of exposure, more sensitive health endpoints, or more susceptible populations would produce more informative epidemiologic studies. Well-reasoned hypotheses suggesting where stronger effects should be found under a causal hypothesis are informative regardless of whether the anticipated larger effects occur. Finding that stronger associations are not found where they would be expected under a causal hypothesis provides meaningful evidence against a causal effect.

Interpreting results from multiple studies can be much more informative than simply generating a pooled measure of association as in routine meta-analysis or tallying the number of positive studies. Studies with varying features that are expected to bear on validity, such as quality of exposure or health outcome assessment or susceptibility to confounding, allow for informative contrasts across studies.^13^ If studies with methodologic features that should yield stronger associations under a causal hypothesis do so, a causal effect is supported, and if they fail to do so, the plausibility of a causal effect is diminished.

Finally, the negative consequences of exaggerating evidence of health risks warrant consideration, no less than understating evidence of harm and missing an opportunity for beneficial action. Overinterpreting the evidence for causal effects of environmental toxicants and health outcomes and claiming they call for regulatory or behavioral change based on precarious evidence reduces the credibility of environmental epidemiology and weakens lines of research that warrant attention and intervention. In the face of declining public trust in science generally^14^ and political exploitation and encouragement of that distrust, we all need to be more cautious in distinguishing between actionable evidence and inconsequential exploitation of data. More than ever, there is a need to address pressing environmental health concerns with rigorous science that will ultimately advance public health. Studies in which the link to environmental exposures is tenuous, the health measure is of unclear importance, and the presence of a causal effect is ambiguous provide little if any progress towards beneficial public health action.

### Conflicts of interest statement

The authors declare that they have no conflicts of interest with regard to the content of this report.
